# *R*-ketamine: a rapid-onset and sustained antidepressant without psychotomimetic side effects

**DOI:** 10.1038/tp.2015.136

**Published:** 2015-09-01

**Authors:** C Yang, Y Shirayama, J-c Zhang, Q Ren, W Yao, M Ma, C Dong, K Hashimoto

**Affiliations:** 1 Division of Clinical Neuroscience, Chiba University Center for Forensic Mental Health, Chiba, Japan; 2 Department of Psychiatry, Teikyo University Chiba Medical Center, Ichihara, Japan

## Abstract

Although the efficacy of racemate ketamine, a rapid onset and sustained antidepressant, for patients with treatment-resistant depression was a serendipitous finding, clinical use of ketamine is limited, due to psychotomimetic side effects and abuse liability. Behavioral and side-effect evaluation tests were applied to compare the two stereoisomers of ketamine. To elucidate their potential therapeutic mechanisms, we examined the effects of these stereoisomers on brain-derived neurotrophic factor (BDNF)–TrkB signaling, and synaptogenesis in selected brain regions. In the social defeat stress and learned helplessness models of depression, *R*-ketamine showed a greater potency and longer-lasting antidepressant effect than *S*-ketamine (esketamine). Furthermore, *R*-ketamine induced a more potent beneficial effect on decreased dendritic spine density, BDNF–TrkB signaling and synaptogenesis in the prefrontal cortex (PFC), CA3 and dentate gyrus (DG) of the hippocampus from depressed mice compared with *S*-ketamine. However, neither stereoisomer affected these alterations in the nucleus accumbens of depressed mice. In behavioral tests for side effects, *S*-ketamine, but not *R*-ketamine, precipitated behavioral abnormalities, such as hyperlocomotion, prepulse inhibition deficits and rewarding effects. In addition, a single dose of *S*-ketamine, but not *R*-ketamine, caused a loss of parvalbumin (PV)-positive cells in the prelimbic region of the medial PFC and DG. These findings suggest that, unlike *S*-ketamine, *R*-ketamine can elicit a sustained antidepressant effect, mediated by increased BDNF–TrkB signaling and synaptogenesis in the PFC, DG and CA3. *R*-ketamine appears to be a potent, long-lasting and safe antidepressant, relative to *S*-ketamine, as *R*-ketamine appears to be free of psychotomimetic side effects and abuse liability.

## Introduction

As well as being the most prevalent, major depressive disorder is also among the most severe and debilitating of the psychiatric illnesses. The World Health Organization estimates that more than 350 million individuals of all ages suffer from depression.^1^ Almost one million lives are lost annually due to suicide, which translates to 3000 deaths daily.^1^ Although antidepressants are generally effective in the treatment of major depressive disorder, it can still take weeks before patients feel the full therapeutic benefit. In addition, approximately two-thirds of these patients fail to respond to pharmacotherapy and for those that do, there is a high rate of relapse.^2^ This highlights the unmet need for therapeutic agents with a rapid onset of action, particularly for patients who do not respond to current antidepressants, many of whom are at an increased risk of suicide.^3^ In this setting, the glutamatergic system represents a promising therapeutic target for major depressive disorder.^4, 5, 6, 7, 8, 9^

Over the past decade, the fast-acting antidepressant effects of the *N*-methyl-d-aspartate (NMDA) receptor antagonist, ketamine, represent one of the most attractive discoveries in the field of psychiatry.^3, 10, 11^ A sub-anesthetic dose (0.5 mg kg^−1^) of ketamine confers a rapid antidepressant benefit in depressed patients,^12^ including treatment-resistant depression and treatment-resistant bipolar depression.^13, 14, 15, 16^ Ketamine elicits its rapid antidepressant effect within a couple of hours, and this effect can be sustained for up to 2 weeks in some patients. Crucially, ketamine is reported to exert rapid effects against suicidal ideation.^17, 18, 19^ Recent meta-analyses support the rapid therapeutic action of ketamine in treatment-resistant patients.^20, 21, 22^

Ketamine (or *RS*-ketamine) is a racemic mixture containing equal parts of *R*-ketamine and *S*-ketamine. *S*-ketamine has an approximately fourfold greater affinity for the NMDA receptor than the *R*-ketamine.^10^ Furthermore, *S*-ketamine shows an approximately three- to fourfold greater anesthetic potency and greater undesirable psychotomimetic side effects, compared with *R*-ketamine.^10, 23^ These findings lend weight to the general acceptance that the anesthetic and psychotomimetic actions of ketamine are mediated primarily via the blockade of NMDA receptors.^10, 23, 24^ In the depression model after neonatal dexamethasone exposure, we reported that *R*-ketamine showed greater potency and longer-lasting antidepressant effects than *S*-ketamine,^25^ indicating that *R*-ketamine may be a potent and safe antidepressant relative to *S*-ketamine.^26, 27^

The purpose of this study was to compare the two stereoisomers of ketamine in the social defeat stress and learned helplessness models of depression. In addition, we examined the effects of these stereoisomers on brain-derived neurotrophic factor (BDNF)–TrkB signaling, and synaptogenesis in selected brain regions, as these may be implicated in ketamine's antidepressant effects.^9, 28, 29, 30, 31, 32, 33^ Finally, we examined the action of these stereoisomers on a profile of side effects, including psychotomimetic effects and drug abuse potential.

## Materials and methods

### Animals

Male adult C57BL/6 mice, aged 8 weeks (body weight 20–25 g, Japan SLC, Hamamatsu, Japan) and male adult CD1 (ICR) mice, aged 13–15 weeks (body weight >40 g, Japan SLC) were used for the social defeat stress model. Male Sprague Dawley rats (200–230 g, 7 week olds, Charles River Japan, Tokyo, Japan) were used for the learned helplessness (LH) model. Animals were housed under controlled temperatures and 12-h light/dark cycles (lights on between 0700 and 1900 hours), with *ad libitum* food (CE-2; CLEA Japan, Tokyo, Japan) and water. This study was carried out in strict accordance with the recommendations in the Guide for the Care and Use of Laboratory Animals of the National Institutes of Health. The protocol was approved by the Chiba University Institutional Animal Care and Use Committee.

### Materials

*R*-ketamine hydrochloride and *S*-ketamine hydrochloride were prepared by recrystallization of *RS*-ketamine (Ketalar, ketamine hydrochloride, Daiichi Sankyo Pharmaceutical, Tokyo, Japan) and d-(−)-tartaric acid (or l- (+)-tartaric acid), as described previously.^34^ The purity of these stereoisomers was determined by a high-performance liquid chromatography (CHIRALPAK IA, column size: 250 × 4.6 mm, mobile phase: *n*-hexane/dichloromethane/diethylamine (75/25/0.1), Daicel, Tokyo, Japan). NBQX, 2,3-dioxo-6-nitro-1,2,3,4-tetrahydrobenzo[*f*]quinoxaline-7-sulfonamide (catalog number: 0373, Tocris Bioscience, Bristol, UK, 10 mg kg^−1^) was dissolved in saline. ANA-12, N2-(2-{[(2-oxoazepan-3-yl) amino]carbonyl}phenyl)benzo[b]thiophene-2-carboxamide (catalog number: BTB06525SC, Maybridge, Trevillett Tintagel, Cornwall, UK, 0.5 mg kg^−1^) was prepared in vehicle of 1% dimethylsulfoxide in phosphate-buffered saline. The dose of ketamine, NBQX and ANA-12 was selected as reported previously.^34, 35, 36, 37, 38, 39, 40^ Other reagents were purchased commercially.

### Social defeat stress model

The procedure of social defeat stress was performed as previously reported.^41, 42^ Every day the C57BL/6 mice were exposed to a different CD1 aggressor mouse for 10 min, total for 10 days. When the social defeat session ended, the resident CD1 mouse and the intruder mouse were housed in one half of the cage separated by a perforated Plexiglas divider to allow visual, olfactory and auditory contact for the remainder of the 24- h period. At 24 h after the last session, all mice were housed individually. On day 11, a social avoidance test was performed to identify subgroups of mice that were susceptible and unsusceptible to social defeat stress. This was accomplished by placing mice in an interaction test box (42 × 42 cm) with an empty wire-mesh cage (10 × 4.5 cm) located at one end. The movement of the mice was tracked for 2.5 min, followed by 2.5 min in the presence of an unfamiliar aggressor confined in the wire-mesh cage. The duration of the subject's presence in the 'interaction zone' (defined as the 8-cm-wide area surrounding the wire-mesh cage) was recorded by a stopwatch. The interaction ratio was calculated as the time spent in an interaction zone with an aggressor/time spent in an interaction zone without an aggressor. An interaction ratio of 1 was set as the cutoff: mice with scores <1 were defined as 'susceptible' to social defeat stress and those with scores ⩾1 were defined as 'unsusceptible'.^42^ Only susceptible mice were used in the experiments.

### Learned helplessness stress model

Learned helplessness (LH) paradigm was performed as reported previously.^36, 43, 44^ To create the LH paradigm, rats are initially exposed to uncontrollable stress. When the rat is later placed in a situation in which shock is controllable (escapable), it not only fails to acquire the escape responses, but also often makes no efforts to escape the shock at all. LH behavioral tests were performed using the Gemini Avoidance System (San Diego Instruments, San Diego, CA, USA). This apparatus was divided into two compartments by a retractable door. On days 1 and 2, rats were subjected to 30 inescapable electric foot shock (0.65 mA, 30 s duration, at random intervals (mean 30 s, average 18–42 s)). On day 3, a two-way conditioned avoidance test was performed as a post-shock test to determine whether the rats would show the predicted escape deficits. This screening session consisted of 30 trials in which electric foot shocks (0.65 mA, 6 s duration, at random intervals (mean 30 s, average 18–42 s)) were preceded by a 3-s conditioned stimulus tone that remained on until the shock was terminated. Subsequently, saline (1 ml kg^−1^), *R*-ketamine (20 mg kg^−1^) or *S*-ketamine (20 mg kg^−1^) was administered intraperitoneally into control and LH rats. On day 8, a two-way conditioned avoidance test was performed. This test session consisted of 30 trials in which electric foot shocks (0.65 mA, 30 s duration, at random intervals (mean 30 s, average 18–42 s)) were preceded by a 3-s conditioned stimulus tone that remained on until the shock was terminated. The numbers of escape failures and the latency to escape in each of the 30 trials were recorded by the Gemini Avoidance System (San Diego Instruments).

### Behavioral tests of antidepressant effects

Behavioral tests of antidepressant effects including locomotion, tail suspension test, forced swimming test and sucrose preference test were performed as reported previously (for details, see Supplementary Information).^34, 37^

### Behavioral tests of side effects

Behavioral tests of side effects such as locomotion, prepulse inhibition (PPI) test and conditioned place preference (CPP) test were performed as reported previously (for details, see Supplementary Information).^45, 46, 47^

### Golgi staining

Golgi staining was performed using the FD Rapid GolgiStain Kit (FD Neuro Technologies, Columbia, MD, USA), following the manufacturer's instructions (for details, see Supplementary Information).

### Western blot analysis

Western blot analysis was performed as reported previously (for details, see Supplementary Information).^34, 36^

### Parvalbumin immunohistochemistry

Parvalbumin (PV) immunohistochemistry was performed as previously reported (for details, see Supplementary Information).^48^

### Statistical analysis

The data are shown as the mean±s.e.m. Analysis was performed using PASW Statistics 20 (formerly SPSS Statistics; SPSS, Tokyo, Japan). Comparisons between groups were performed using the one-way analysis of variance (ANOVA) or two-way ANOVA, followed by a *post hoc* Fisher's least significant difference test. The PPI data were analyzed using multivariate analysis of variance, followed by a *post hoc* Fisher's least significant difference test. The *P*-values of less than 0.05 were considered statistically significant.

## Results

### *R*-ketamine shows greater potency and longer-lasting antidepressant effects than *S*-ketamine in the social defeat stress model

**Figure 1:**
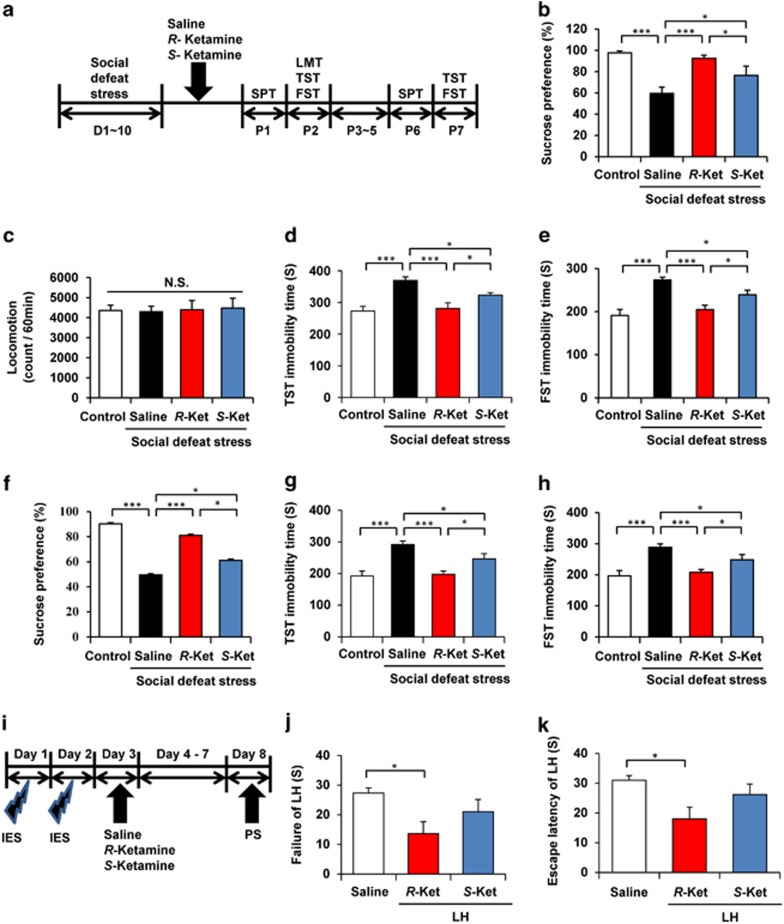
Antidepressant effects of *R*-ketamine and *S*-ketamine in social defeat stress and LH models of depression. (**a**) The schedule of social defeat stress model and behavioral tests after treatment. (**b**) One percent SPT was performed 1 day after a single dose of saline, *R*-ketamine (10 mg kg^−1^) or *S*-ketamine (10 mg kg^−1^) (one-way ANOVA, F_3,42_=11.05, *P*=0.002). (**c**–**e**) Behavioral tests, including LMT (F_3,35_=0.038, *P*=0.99), TST (F_3,34_=12.046, *P*<0.001) and FST (F_3,34_=13.235, *P*<0.001), were performed 2 days after a single dose. (**f**) SPT was performed 6 days after a single dose (F_3,34_=9.974, *P*<0.001). (**g**, **h**) TST (F_3,32_=12.019, *P*<0.001) and FST (F_3,32_=14.479, *P*<0.001) were performed 7 days after a single dose. Data are shown as mean±s.e.m. (*n*=8–11). *P<0.05, and ***P<0.001. (**i**) The schedule of learned helplessness (LH) model and behavioral tests after treatment. (**j**, **k**) The failure of LH (one-way ANOVA, F_2,15_=3.903, *P*=0.043) and the escape latency of LH (F_2,15_=4.317, *P*=0.033) were measured 5 days after a single dose of saline, *R*-ketamine (20 mg kg^−1^) or *S*-ketamine (20 mg kg^−1^). Data are shown as mean±s.e.m. (*n*=6). *P<0.05. ANOVA, analysis of variance; FST, forced swimming test; IES, inescapable electric foot shock; LH, learned helplessness; LMT, locomotion test; NS, not significant; PS, post-shock test; SPT, sucrose preference test; TST, tail suspension test.

First, we examined the effects of *R*-ketamine (10 mg kg^−1^) and *S*-ketamine (10 mg kg^−1^) on the social defeat stress model of depression (Figure 1a). Both stereoisomers of ketamine significantly attenuated the reduced sucrose preference seen in depressed mice, one day after a single dose. The anti-anhedonia effect of *R*-ketamine was significantly more potent than that of *S*-ketamine (Figure 1b). There were no differences in locomotion among the four groups (Figure 1c). In the tail suspension test and forced swimming test, both *R*-ketamine and *S*-ketamine significantly reduced the typically increased immobility time displayed by depressed mice, 2 days after a single dose. The antidepressant effect of *R*-ketamine was also significantly more potent relative to that of *S*-ketamine (Figures 1d and e). Anti-anhedonia and antidepressant effects of *R*-ketamine were significantly more potent than those of *S*-ketamine, although both stereoisomers showed antidepressant effects 6–7 days after a single dose (Figures 1f–h). These data suggest a greater therapeutic potency for *R*-ketamine in the social defeat stress model.

### Antidepressant effects of *R*-ketamine, but not *S*-ketamine, in the rat LH model

In the rat LH model of depression, vehicle (10 ml kg^−1^), *R*-ketamine (20 mg kg^−1^) or *S*-ketamine (20 mg kg^−1^) were administered intraperitoneally into rats. Behavioral tests were performed 5 days after a single dose of test compound (Figure 1i). *R*-ketamine, but not *S*-ketamine, significantly (*P*<0.05) attenuated the failure of LH and escape latency of LH (Figures 1j and k). These data imply an antidepressant effect for *R*-ketamine, but not *S*-ketamine in the rat LH model.

### Effects of *R*-ketamine and *S*-ketamine on alterations in brain dendritic spine density induced by social defeat stress

**Figure 2:**
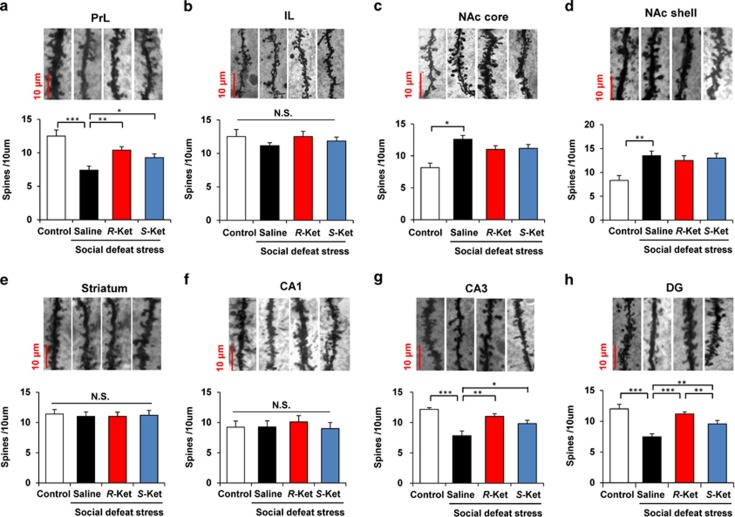
Effects of *R*-ketamine and *S*-ketamine on alterations in dendritic spine density in the brain regions after social defeat stress. Brain samples were collected 8 days after a single dose of saline, *R*-ketamine (10 mg kg^−1^) or *S*-ketamine (10 mg kg^−1^) for Golgi–Cox staining. Representative photomicrographs of Golgi–Cox-stained pyramidal neurons in the PrL of mPFC, IL of mPFC, NAc core, NAc shell, striatum, CA1, CA3 and DG of hippocampus from animals of each group. Scale bar=10 μm. (**a**) PrL (one-way analysis of variance, F_3,20_=8.123, *P*=0.001); (**b**) IL (F_3,20_=0.528, *P*=0.668); (**c**) NAc core (F_3,20_=6.318, *P*=0.003); (**d**) NAc shell (F_3,20_=6.332, *P*=0.003); (**e**) striatum (F_3,20_=0.381, *P*=0.768); (**f**) CA1 (F_3,20_=0.459, *P*=0.714); (**g**) CA3 (F_3,20_=8.448, *P*=0.001); (**h**) DG (F_3,20_=5.546, *P*=0.006). Values represent the mean±s.e.m. (*n*=6). *P<0.05, **P<0.01, ***P<0.001. DG, dentate gyrus; IL, infralimbic region; mPFC, medial prefrontal cortex; NAc, nucleus accumbens; N.S., not significant; PrL, prelimbic region.

Previous reports demonstrated that the depression-like phenotype brought on by stress and inflammation causes alterations in dendritic spine density in the medial prefrontal cortex (mPFC), CA3 and dentate gyrus (DG) of the hippocampus and nucleus accumbens (NAc).^34, 35, 36^ In this study, we examined whether the two stereoisomers could affect alterations in the dendritic spines of the prelimbic (PrL) and infralimbic (IL) regions of mPFC, shell and core of the NAc, striatum, and CA1, CA3 and DG of the hippocampus, after social defeat stress. We found significant differences in the PrL of mPFC, NAc core, NAc shell, CA3 and DG, but not IL of the mPFC, striatum and CA1 (Figure 2). Social defeat stress significantly decreased dendritic spine density in the PrL of mPFC (*P*<0.001), CA3 (*P*<0.001) and DG (*P*<0.001). Both *R*-ketamine (10 mg kg^−1^) and *S*-ketamine (10 mg kg^−1^), significantly attenuated the reduced spine density typically seen in the PrL, CA3 and DG from depressed mice, 8 days after a single dose (Figures 2a, g and h). Interestingly, in the DG, *R*-ketamine conferred a significantly (*P*<0.01) more potent effect than *S*-ketamine (Figure 2h). In contrast, social defeat stress significantly increased dendritic spine density in the NAc core (*P*<0.05) and NAc shell (*P*<0.01) (Figures 2c and d). Neither *R*-ketamine nor *S*-ketamine altered spine density in the NAc of depressed mice. These findings implicate the PrL of mPFC, CA3 and DG, but not NAc, in the mechanistic action of ketamine, as synaptogenesis is thought to be involved in the action of antidepressants.

### Role of AMPA receptors and BDNF–TrkB signaling in the antidepressant action of ketamine isomers

**Figure 3:**
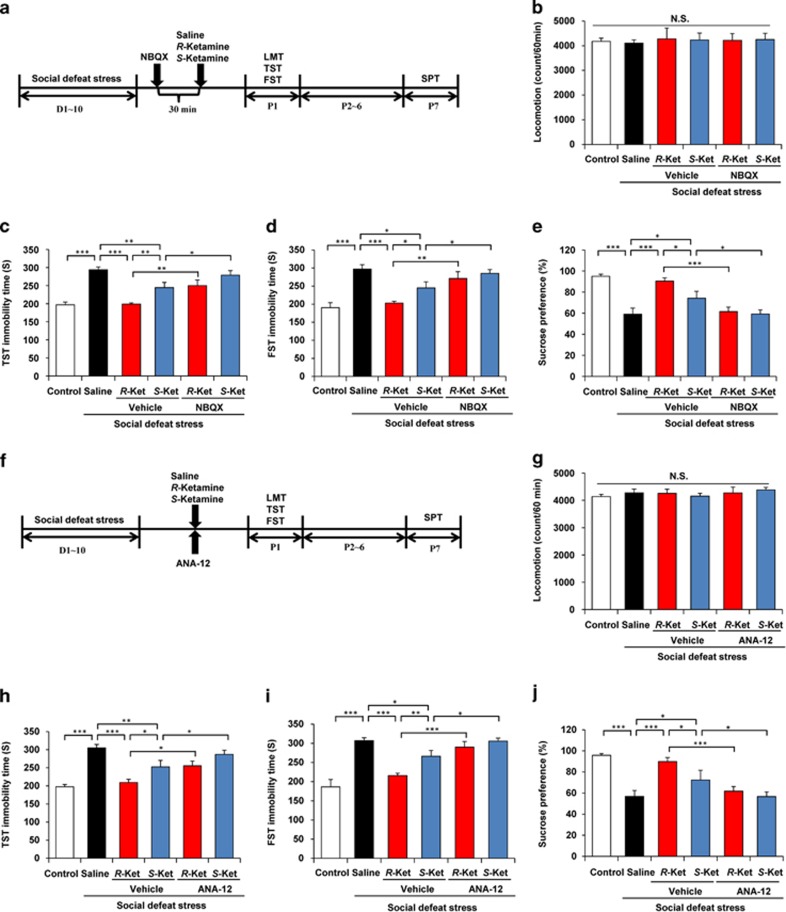
Role of AMPA receptor and BDNF–TrkB signaling in the mechanisms of antidepressant effect for *R*-ketamine and *S*-ketamine. (**a**) The schedule of social defeat stress model and behavioral tests after treatment. NBQX (10 mg kg^−1^), an AMPA receptor antagonist, was administered 30 min before saline, *R*-ketamine (10 mg kg^−1^) or *S*-ketamine (10 mg kg^−1^). Behavioral tests, including (**b**) LMT (one-way ANOVA, F_5,34_=0.06, *P*=0.997), (**c**) TST (F_5,39_=14.628, *P*<0.001) and (**d**) FST (F_5,42_=9.015, *P*<0.001), were performed 1 day after a single dose. (**e**) SPT was 7 days after a single dose of saline, *R*-ketamine (10 mg kg^−1^) or *S*-ketamine (10 mg kg^−1^) (F_5,40_=11.748, *P*<0.001). Values represent the mean±s.e.m. (*n*=6–9). *P<0.05, **P<0.01 and ***P<0.001. (**f**) The schedule of social defeat stress model and behavioral tests after treatment. ANA-12 (0.5 mg kg^−1^), a TrkB antagonist, was co-administered with saline or *R*-ketamine (10 mg kg^−1^) or *S*-ketamine (10 mg kg^−1^). Behavioral tests, including (**g**) LMT (one-way ANOVA, F_5,37_=0.414, *P*=0.836), (**h**) TST (F_5,33_=14.044, *P*<0.001) and (**i**) FST (F_5,32_=15.783, *P*<0.001), were performed 1 day after a single dose. (**j**) The SPT was 7 days after a single dose of saline, *R*-ketamine or *S*-ketamine (F_5,36_=11.825, *P*<0.001). Values represent the mean±s.e.m. (*n*=6–8). *P<0.05, **P<0.01 and ***P<0.001. AMPA, α-amino-3-hydroxy-5-methyl-4-isoxazolepropionic acid receptor; ANOVA, analysis of variance; BDNF, brain-derived neurotrophic factor; FST, forced swimming test; LMT, locomotion test; N.S., not significant; SPT, sucrose preference test; TST, tail suspension test.

As the α-amino-3-hydroxy-5-methyl-4-isoxazolepropionic acid (AMPA) receptor has a role in ketamine's antidepressant activity,^49^ we examined the effects of NBQX, an AMPA receptor antagonist, on antidepressant effects of *R*-ketamine and *S*-ketamine (Figure 3a). Pretreatment with NBQX (10 mg kg^−1^, intraperitoneally, 30 min) significantly blocked the antidepressant effects of *R*-ketamine (10 mg kg^−1^) and *S*-ketamine (10 mg kg^−1^) in the social defeat stress model (Figures 3c and e). These findings highlight a possible role for AMPA receptors in the antidepressant mechanisms of both stereoisomers.

As BDNF–TrkB signaling is a putative pathway in ketamine's therapeutic mode of action,^31, 33^ we examined the effects of ANA-12, a TrkB antagonist, on the activity of *R*- and *S*-ketamine (Figure 3f). Co-treatment with ANA-12 (0.5 mg kg^−1^, intraperitoneally) significantly blocked the antidepressant effects of *R*-ketamine (10 mg kg^−1^) and *S*-ketamine (10 mg kg^−1^) in the social defeat stress model (Figures 3h and j). These findings place BDNF–TrkB signaling in the mechanistic pathways of both stereoisomers.

### Effects of *R*-ketamine and *S*-ketamine on alterations in BDNF, TrkB phosphorylation and synaptogenesis in selected brain regions

**Figure 4:**
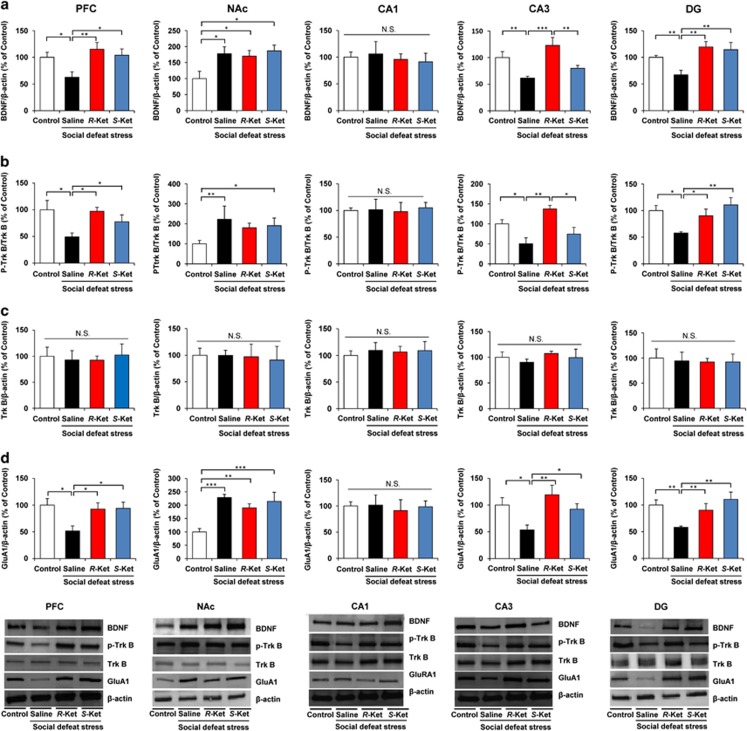
Effect for *R*-ketamine and *S*-ketamine on the BDNF, TrkB phosphorylation and GluA1 in the brain regions. Brain samples were collected 7 days after a single dose of saline, *R*-ketamine (10 mg kg^−1^) or *S*-ketamine (10 mg kg^−1^) for Golgi–Cox staining. (**a**) Western blot analysis of BDNF in PFC, NAc, CA1, CA3 and DG. PFC (one-way ANOVA, F_3,13_=5.759, *P*=0.009); NAc (F_3,13_=3.544, *P*=0.045); CA1 (F_3,14_=0.065, *P*=0.978); CA3 (F_3,13_=8.324, *P*=0.002); DG (F_3,13_=7.444, *P*=0.003). The value was expressed as a percentage of that of control mice. Values represent the mean±s.e.m. (*n*=5 or 6). *P<0.05, **P<0.01, ***P<0.001. (**b**, **c**) Effects of *R*-ketamine and *S*-ketamine on changes in phosphorylation of TrkB in the mouse brain after social defeat stress. The ratio of p-TrkB to total TrkB in the brain regions was shown. PFC (one-way ANOVA, F_3,13_=3.962, *P*=0.033); NAc (F_3,13_=3.613, *P*=0.043); CA1 (F_3,14_=0.05, *P*=0.984); CA3 (F_3,14_=6.545, *P*=0.005); DG (F_3,13_=7.612, *P*=0.003). Total levels of TrkB protein in the all regions were not different among the four groups. Values represent the mean±s.e.m. (*n*=5 or 6). *P<0.05, **P<0.01. (**d**) Western blot analysis of GluA1 in PFC, NAc, CA1, CA3 and DG of the hippocampus. PFC (one-way ANOVA, F_3,13_=3.619, *P*=0.04); NAc (F_3,14_=7.834, *P*=0.003); CA1 (F_3,14_=0.060, *P*=0.98); CA3 (F_3,14_=5.207, *P*=0.013); DG (F_3,14_=5.108, *P*=0.014). The value was expressed as a percentage of that of control mice. Values represent the mean±s.e.m. (*n*=5 or 6). *P<0.05, **P<0.01, ***P<0.001. ANOVA, analysis of variance; BDNF, brain-derived neurotrophic factor; DG, dentate gyrus; NAc, nucleus accumbens; N.S., not significant; PFC, prefrontal cortex.

We performed western blot analyses of BDNF, TrkB, phosphorylated TrkB (p-TrkB) and GluA1 in selected brain regions (PFC, NAc, DG, CA1 and CA3 of the hippocampus), 7 days after a dose of saline, *R*-ketamine (10 mg kg^−1^) or *S*-ketamine (10 mg kg^−1^). Social defeat stress significantly decreased levels of BDNF protein in the PFC, DG and CA3, but not CA1, while significantly increasing BDNF protein in the NAc. Interestingly, both stereoisomers significantly attenuated reduced levels of BDNF protein in the PFC, CA3 and DG after social defeat stress, although neither affected the increased levels of BDNF protein in the NAc (Figure 4a). Effects of *R*-ketamine on the reduction of BDNF in CA3 were significantly more potent than *S*-ketamine (Figure 4a).

To clarify the role of TrkB phosphorylation in the action of the stereoisomers, we performed western blot analyses of TrkB and p-TrkB, an activated form of TrkB, in samples from PFC, NAc and hippocampus (CA1, CA3 and DG). Tissue levels of TrkB in the all tested regions did not differ among the four groups (Figure 4c). Social defeat stress significantly decreased the ratio of p-TrkB/TrkB protein in the PFC, CA3 and DG, but not CA1, while significantly increasing the ratio of p-TrkB/TrkB protein in the NAc (Figure 4b). Furthermore, *R*-ketamine significantly attenuated the reduced ratio of p-TrkB/TrkB protein in PFC, CA3 and DG. *R*-ketamine conferred a significantly greater effect on the ratio of p-TrkB/TrkB protein in the CA3 and DG relative to *S*-ketamine (Figure 4b). In contrast, *R*-ketamine and *S*-ketamine did not alter the increased ratio of p-TrkB/TrkB protein in NAc. These findings suggest that BDNF–TrkB signaling in PFC and hippocampus (CA3 and DG) is integral to the mechanisms of both stereoisomers.

Protein levels of GluA1, a subtype of the AMPA receptor, are a marker for synaptogenesis.^9, 32^ Social defeat stress significantly decreased the levels of GluA1 in PFC, CA3 and DG, but not CA1, while significantly increasing GluA1 protein in the NAc (Figure 4d). Furthermore, both stereoisomers significantly attenuated the reduction of GluA1 in PFC, CA3 and DG. Both *R*- and *S*-ketamine produced similar effects on GluA1 protein in the PFC, CA3 and DG (Figure 4d). In contrast, *R*-ketamine and *S*-ketamine did not alter the increased levels of GluA1 in NAc (Figure 4d).

### Behavioral side effects of *R*-ketamine and *S*-ketamine

**Figure 5:**
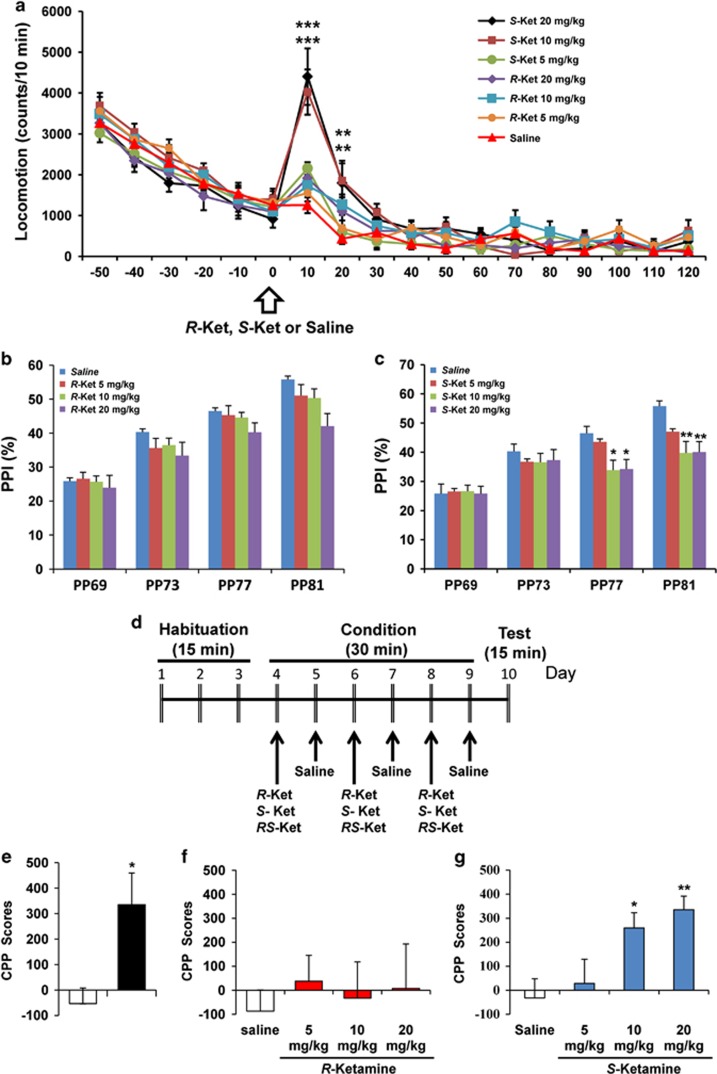
Side-effect profiles for *S*-ketamine, but not *R*-ketamine, in mice. (**a**) Effects of *R*-ketamine and *S*-ketamine on locomotion in control mice. One hour after habituation, saline, *R*-ketamine (5, 10 or 20 mg kg^−1^) or *S*-ketamine (5, 10 or 20 mg kg^−1^) was administered intraperitoneally into mice. Two-way ANOVA analysis revealed significant interactions (drug: F_6,125_=6.441, *P*<0.001; time: F_17,125_=138.838, *P*<0.001; interaction (drug × time): F_102,1125_=2.814, *P*<0.001). Values represent the mean±s.e.m. (*n*=8). **P<0.01, ***P<0.001 compared with the saline-treated group. (**b**) Effects of *R*-ketamine (5, 10 or 20 mg kg^−1^) on PPI test in control mice. The MANOVA revealed no significant effect (Wilks' lambda=0.713, *P*=0.333). Values represent the mean±s.e.m. (*n*=8). (**c**) Effects of *S*-ketamine (5, 10 or 20 mg kg^−1^) on the PPI test in control mice. The MANOVA revealed significant effect (Wilks' lambda=0.554, *P*=0.019). Values represent the mean±s.e.m. (*n*=8). *P<0.05, **P<0.01 compared with the saline-treated group. (**d**) The schedule of CPP model and behavioral tests after treatment. (**e**) *RS*-ketamine (10 mg kg^−1^) significantly (*P*=0.0125) increased CPP scores in mice (*n*=9). *P<0.05 compared with the saline-treated group. (**f**) *R*-ketamine (5, 10 or 20 mg kg^−1^) did not increase CPP score (F_3,35_=0.147, *P*=0.931). (**g**) *S*-Ketamine (5, 10 or 20 mg kg^−1^) significantly increased CPP score (F_3,34_=5.441, *P*=0.004). Values represent the mean±s.e.m. (*n*=9–10). *P<0.05, **P<0.01 compared with the saline-treated group. ANOVA, analysis of variance; CPP, conditioned place preference test; MANOVA, multivariate analysis of variance; PPI, prepulse inhibition.

First, we examined the effects of both stereoisomers on spontaneous locomotion in control mice. A single dose of *S*-ketamine (5, 10 or 20 mg kg^−1^) elevated locomotion in mice, in a significant and dose-dependent manner, but the hyperactivity quickly returned to control levels. In contrast, an administration of *R*-ketamine (5, 10 or 20 mg kg^−1^) had no effect on locomotion in mice (Figure 5a).

Next, we examined the effects of the stereoisomers on PPI in control mice. A single administration of *R*-ketamine (5, 10 or 20 mg kg^−1^) did not alter PPI in the mice (Figure 5b). In contrast, a dose of *S*-ketamine (10 and 20 mg kg^−1^) caused significant PPI deficits at 77 dB and 81 dB levels (Figure 5c). These findings suggest that, unlike *R*-ketamine, *S*-ketamine may be more prone to cause psychotomimetic side effects.

The recreational use of ketamine has increased in many parts of the world, and its abuse is often associated with harmful physical and psychological consequences.^50^ In this study, we examined the rewarding effects of *RS*-ketamine (racemate) and the two stereoisomers under the CPP test (Figure 5d). *RS*-Ketamine (10 mg kg^−1^) significantly increased CPP scores in mice (Figure 5e), whereas *R*-ketamine (5, 10 or 20 mg kg^−1^) had no effect on CPP scores (Figure 5f). In contrast, *S*-ketamine (5, 10 or 20 mg kg^−1^) significantly increased CPP scores, in a dose-dependent manner (Figure 5g). The data suggest that *S*-ketamine and *RS*-ketamine, but not *R*-ketamine, may induce rewarding effects.

### Effects of *R*-ketamine and *S*-ketamine on PV-positive cells in the brain

**Figure 6:**
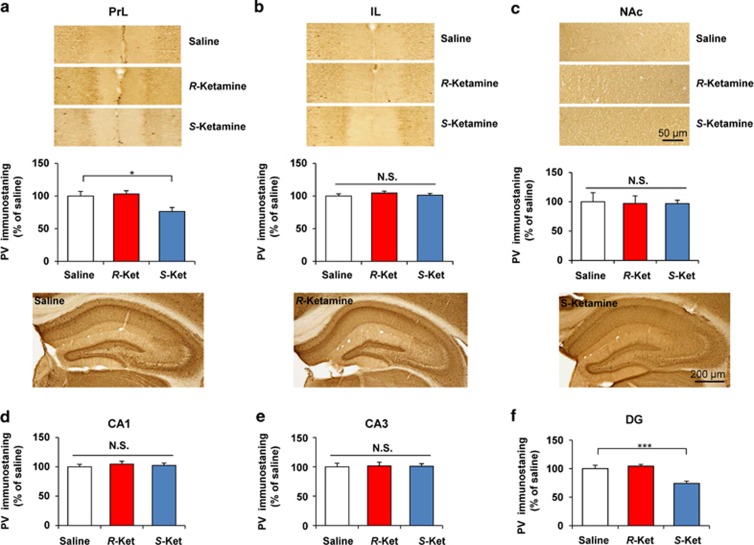
Effect of *R*-ketamine and *S*-ketamine on PV-positive immunostaining in the brain. Mice were perfused 30 min after a single dose of saline, *R*-ketamine (10 mg kg^−1^) or *S*-ketamine (10 mg kg^−1^). Then PV immunohistochemistry was performed. Representative photomicrographs of PV immunohistochemistry in the PrL (**a**) and IL (**b**) of mPFC, NAc (**c**), CA1 (**d**), CA3 (**e**) and DG (**f**) of hippocampus from animals of each group. Scale bar=10 μm (**a**–**c**) or 200 μm (**d**–**f**). One-way analysis of variance revealed significant effects in the PrL (F_2,14_=7.57, *P*=0.016) of mPFC and DG (F_2,15_=13.834, *P*<0.001), but not IL of mPFC (F_2,14_=0.602, *P*=0.562), NAc (F_2,15_=0.019, *P*=0.981), CA3 (F_2,15_=0.015, *P*=0.985) and CA1 (F_2,14_=0.234, *P*=0.795). The data show the mean±s.e.m. (*n*=5 or 6). *P<0.05, ***P<0.001 compared with the saline-treated group. DG, dentate gyrus; IL, infralimbic region; mPFC, medial prefrontal cortex; NAc, nucleus accumbens; N.S., not significant; PrL, prelimbic region; PV, parvalbumin.

A reduction in PV-positive cells in the brain is associated with psychosis and cognitive impairment in schizophrenia.^51, 52^ A single administration of *R*-ketamine (10 mg kg^−1^, 30 min) had no effect on the proportion of PV-positive cells in any of the tested brain regions (Figures 6a and f). In contrast, a single dose of *S*-ketamine (10 mg kg^−1^, 30 min) significantly decreased the proportion of PV-positive cells in the PrL region of the mPFC (Figure 6a, *P*=0.006) and DG of the hippocampus (Figure 6f, *P*<0.001), but not in other regions (IL region of mPFC, NAc, CA1 and CA3) (Figures 6b and e).

## Discussion

In this study, we found *R*-ketamine to be a more potent and longer-lasting antidepressant than *S*-ketamine. The superior potency of *R*-ketamine's effects over *S*-ketamine was demonstrated in the social defeat stress model of depression. Furthermore, *R*-ketamine showed antidepressant effects in the rat LH model of depression, whereas *S*-ketamine showed none. To the best of our knowledge, this is the first report comparing the antidepressant effects of the ketamine stereoisomers in the social defeat stress and LH models of depression. These findings suggest that *R*-ketamine is a more efficacious antidepressant than *S*-ketamine.

Accumulating evidence suggests that BDNF–TrkB signaling is key to the depression-like phenotype.^53, 54, 55, 56^ In this study, we found a marked reduction of BDNF protein in the PFC, DG and CA3, but not CA1, of depressed mice after social defeat stress, consistent with recent results from the inflammation model and rat LH model.^34, 36^ In contrast, we also found that social defeat stress induced a marked increase in BDNF protein within the NAc, also in keeping with recent papers on the inflammation and LH models.^34, 36^ Furthermore, we found that social defeat stress caused decreased phosphorylation of TrkB in the PFC, CA3 and DG, and increased phosphorylation of TrkB in the NAc. Thus, it is probable that social defeat stress promoted decreased BDNF–TrkB signaling in PFC and hippocampus (CA3 and DG), while increasing signals in the NAc, inducing depression-like behavior in rodents. This study also highlighted that both *R*-ketamine and *S*-ketamine significantly attenuated reduced BDNF level in the PFC, CA3 and DG, although these drugs did not affect the increased BDNF levels in the NAc of depressed mice. Interestingly, ANA-12, a TrkB antagonist, was able to block the antidepressant effects of both stereoisomers, suggestive of a role for BDNF–TrkB signaling in ketamine's antidepressant mechanism. Given the role of BDNF–TrkB signaling in the depression-like phenotype,^53, 54, 55, 56^ it is likely that both stereoisomers exert antidepressant effects by normalizing BDNF levels in the PFC and hippocampus (CA3 and DG), but not NAc. Further detailed studies examining the effects of ketamine on BDNF–TrkB signaling are necessary.

Tracking dendritic morphology, we detected opposing changes in spine density between the mPFC, hippocampus and NAc. The reduced spine density detected in the PrL of mPFC, CA3 and DG, is similar to the findings seen in rodents in the unpredictable chronic mild stress, inflammation and LH models.^34, 36, 57^ In contrast, we found increased spine density in the NAc of depressed mice under social defeat stress, consistent with results from the inflammation, and LH models.^34, 36^ Interestingly, both stereoisomers of ketamine attenuated the reduction in spine density in the PrL, but not the IL region, of mPFC, CA3 and DG, although they did not alter increased spine density in the core and shell of the NAc. A recent electrophysiology study demonstrated that ketamine enhanced corticotropin-releasing hormone-induced excitatory postsynaptic currents in conjunction with an increased density of basal dendritic synaptic spines in the PrL, but not IL, of mPFC.^58^ In contrast, Fuchikami *et al.* reported that neuronal inactivation of the IL completely blocked the antidepressant effects of systemic ketamine in the rodent model, and that ketamine microinjection of IL reproduced antidepressant effects.^59^ Considering that synaptogenesis is a key function in the action of antidepressants,^9, 30, 32^ it is likely that the PrL region of mPFC, CA3 and DG, but not the IL region of mPFC and NAc, are involved in this drug's action.

The pharmacokinetic profile of ketamine in male C57/B6 mice has previously been reported. Its half-life in mouse plasma is ~30 min,^60^ indicating a possible rapid clearance from the body. The two stereoisomers of ketamine share similar pharmacokinetic profiles.^10^ Therefore, it is unlikely that the differential antidepressant effects noted here between *R*- and *S*-ketamine are due to differences in their pharmacokinetic profiles.

*S*-ketamine (Ki=0.30 μm) has an approximately fourfold greater affinity for the NMDA receptor than the *R*-isomer (Ki=1.40 μm).^10, 61^ It is therefore unlikely that this receptor has a major role in the long-lasting effects of *R*-ketamine, although antagonism at this receptor may promote its rapid action. Possible interactions with other systems such as opioid receptors, sigma-1 receptor chaperones or voltage-dependent ion channels may also mediate the antidepressant effects of ketamine.^5, 10, 23^ Nonetheless, further detailed studies on the molecular basis of *R*-ketamine's actions are needed.

Clinical use of ketamine is limited due to its side effects. Unlike *S*-ketamine, *R*-ketamine does not appear to cause psychotomimetic effects, based on the lack of behavioral abnormalities (for example, hyperlocomotion and PPI deficits) observed in rodents after treatment. A randomized study in healthy male volunteers (*N*=10) showed that subjective side effects were more pronounced for *S*-ketamine than *R*-ketamine.^62^ A positron emission tomography study in healthy volunteers demonstrated that psychotomimetic doses of *S*-ketamine markedly increased cerebral metabolic rates of glucose in the frontal cortex and thalamus.^63^ In contrast, equimolar doses of *R*-ketamine tended to decrease cerebral metabolic rates of glucose across the brain, producing no psychotic symptoms, but instead, a state of relaxation and well being.^63^ Thus, it would appear that the psychotomimetic and hyperfrontal metabolic actions of ketamine are mainly induced by its *S*-isomer.^63^

Given that the loss of PV-positive cells contributes to the pathogenesis of schizophrenia,^51^ it is unlikely that *R*-ketamine induces psychotomimetic side effects in humans since, unlike *S*-ketamine, *R*-ketamine did not cause a reduction of PV-positive cells in the brain. Repeated use of ketamine can result in drug abuse in humans.^50^ In CPP tests, *R*-ketamine did not increase CPP scores in mice, whereas *RS*-ketamine (racemate) and *S*-ketamine did so in a significant manner. Combined data points to *R*-ketamine being free of psychotomimetic side effects and abuse potential in humans.

In conclusion, our study shows that a single dose of either of the two stereoisomers of ketamine can produce rapid antidepressant effects in the social defeat stress model of depression. However, unlike *S*-ketamine, *R*-ketamine can elicit a sustained antidepressant effect in this rodent model. Furthermore, it is likely that increased synaptogenesis in the PrL region of the mPFC, DG and CA3 of the hippocampus may mediate the sustained antidepressant response of *R*-ketamine. Finally, *R*-ketamine appears to be a potent, long-lasting and safe antidepressant, relative *S*-ketamine, as *R*-ketamine is free of psychotomimetic side effects and abuse liability.
