# Correction: *R*-ketamine: a rapid-onset and sustained antidepressant without psychotomimetic side effects

**DOI:** 10.1038/s41398-020-00983-3

**Published:** 2020-08-21

**Authors:** C. Yang, Y. Shirayama, J-c Zhang, Q. Ren, W. Yao, M. Ma, C. Dong, K. Hashimoto

**Affiliations:** 1 Division of Clinical Neuroscience, Chiba University Center for Forensic Mental Health, Chiba, Japan; 2 Department of Psychiatry, Teikyo University Chiba Medical Center, Ichihara, Japan

Correction to: *Translational Psychiatry*

10.1038/tp.2015.136 published online 01 September 2015

In the original Article, Figs. 2 and 4 were incorrect due to a duplication error. We cannot update the original Article as we no longer use the software on which it was processed, so we are providing the correct figures here in this correction notice.

**Fig. 2:**
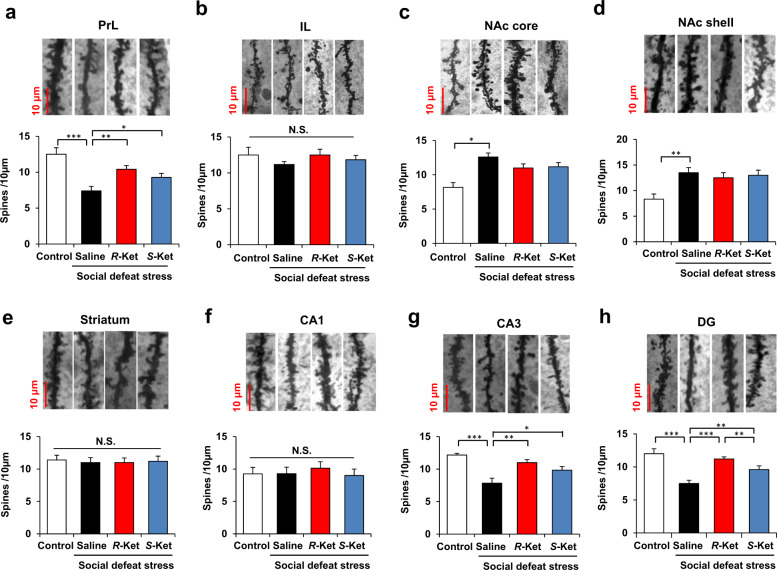
▓

**Fig. 4:**
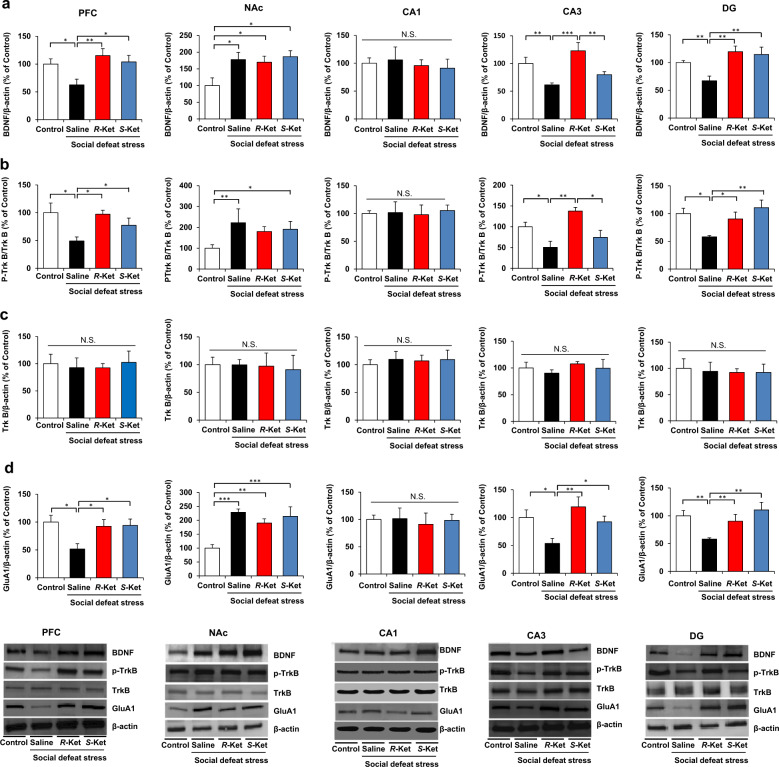
▓

