# Supplementary material for: R-ketamine: a rapid-onset and sustained antidepressant without psychotomimetic side effects
Source: Transl Psychiatry. 2015 Sep 1;5(9):e632–. doi: 10.1038/tp.2015.136 (PMC5068814; doi:10.1038/tp.2015.136)
Supplement: Supplementary Information [file tp2015136x1.docx]

**Supporting Information**

**SI Materials and Methods**

*Behavioral tests of antidepressant effects*

Behavioral tests were performed as reported previously (34, 37). Locomotion: the locomotor activity was measured by an animal movement analysis system SCANETMV-40 (MELQUEST Co., Ltd., Toyama, Japan), the mice were placed in experimental cages (length × width × height: 560 × 560 × 330 mm). The cumulative exercise was recorded for 60 minutes. Cages were cleaned between testing session.

Tail suspension test (TST): A small piece of adhesive tape placed approximately 2 cm from the tip of the tail for mouse. A single hole was punched in the tape and mice were hung individually, on a hook. The immobility time was recorded for 10 minutes. Mice were considered immobile only when they hung passively and completely motionless.

Forced swimming test (FST): The FST was tested by an automated forced-swim apparatus SCANETMV-40 (MELQUEST Co., Ltd., Toyama, Japan). The mice were placed individually in a cylinder (diameter: 23 cm; height: 31 cm) containing 15 cm of water, maintained at 23 ± 1℃. Immobility time from activity time as (total) – (active) time was calculated by the apparatus analysis software. The immobility time for mouse was recorded for 6 minutes.

Sucrose preference test (SPT): Mice were exposed to water and 1% sucrose solution for 48 h, followed by 4 hours of water and food deprivation and a 1 hour exposure to two identical bottles, one is water, and another is 1% sucrose solution. The bottles containing water and sucrose were weighed before and at the end of this period and the sucrose preference was determined.

*Behavioral tests of side effects*

Locomotion: After habituation (60 min) in the cage, saline (10 ml/kg), *R*-ketamine (5, 10 or 20 mg/kg), or *S*-ketamine (5, 10 or 20 mg/kg) was injected i.p. into mice. Locomotor activity was measured using an animal movement analysis system (SCANET MV-40, Melquest, Toyama, Japan). The system consisted of a rectangular enclosure (560 x 560 mm). The side walls (height, 60 mm) of the enclosure were equipped with 144 pairs of photosensors located at 6-mm intervals at a height of 30 mm from the bottom edge. An animal was placed in the observation cage 120 minutes from a single dose of saline or ketamine isomers. A pair of photosensors was scanned every 0.1 second to detect the animal’s movements. The intersection of paired photosensors (10 mm apart) in the enclosure was counted as one unit of locomotor activity. Data collected for 180 minutes were used in this study.

Prepulse inhibition (PPI) test: The mice were tested for their acoustic startle reactivity (ASR) in a startle chamber (SR-LAB; San Diego Instruments, San Diego, CA, USA) using the standard methods described previously (45, 46). The test sessions were begun after an initial 10-min acclimation period in the chamber. The mice were subjected to one of six trials: (1) pulse alone, as a 40 ms broadband burst; a pulse (40 ms broadband burst) preceded by 100 ms with a 20 ms prepulse that was (2) 4 dB, (3) 8 dB, (4) 12 dB, or (5) 16 dB over background (65 dB); and (6) background only (no stimulus). The amount of PPI was expressed as the percentage decrease in the amplitude of the startle reactivity caused by presentation of the prepulse (% PPI). Saline (10 ml/kg), or *R*-ketamine (or S-ketamine)(5, 10 or 20 mg/kg) was administered i.p. 20 min (including the 10-min acclimation period) before the machine records. The PPI test lasted 20 min in total.

Conditioned place preference (CPP) test: The place conditioning paradigm (CPP; Brain Science Idea Inc., Osaka, Japan) was used for studying ketamine-induced rewarding effects, as reported previously (47). The test mouse was allowed to move freely between transparent and black boxes for a 15 min session once a day, for 3 days (days 1–3) as preconditioning. On day 3, the time spent in each box was measured. There was no significant difference between time spent in the black compartment with a smooth floor and the white compartment with a textured floor, indicating that there was no place preference before conditioning. On days 4, 6, and 8, saline (10 ml/kg), or *RS*-ketamine (10 mg/kg)(or *R*-ketamine, *S*-ketamine (5, 10 or 20 mg/kg)) was i.p. administered, and then mice were confined to either the transparent or black box for 30 min. On days 5, 7, and 9, mice were given saline and placed in the opposite ketamine-conditioning box for 30 min. On day 10, the post-conditioning test was performed without drug treatment, and the time spent in each box was measured for 15 min. A counterbalanced protocol was used in order to nullify any initial preference by the mouse. The CPP score was designated as the time spent in the drug-conditioning sites, minus the time spent in the saline-conditioning sites.

*Golgi staining*

Golgi staining was performed using the FD Rapid GolgiStain^TM^ Kit (FD Neuro Technologies, Inc., Columbia, MD, USA), following the manufacturer's instructions, as previously reported (34, 36, 60). Mice were deeply anesthetized with sodium pentobarbital, and brains were removed from the skull and rinsed in double distilled water. Brains were immersed in the impregnation solution, made by mixing equal volumes of Solution A and B, overnight and then stored in fresh solution, for 2 weeks in the dark. Brains were transferred into Solution C overnight and then stored in fresh solution at 4°C for 1 week, in the dark. Coronal brain sections (100 µm thickness) were cut on a cryostat (3050S, Leica Microsystems AG, Wetzlar, Germany), with the chamber temperature set at -20°C. Each section was mounted in Solution C, on saline-coated microscope slides. After absorption of excess solution, sections were dried naturally, at room temperature. Dried sections were processed following the manufacturer's instructions. Briefly, images of dendrites within medial prefrontal cortex (mPFC), hippocampal CA1, CA3, and dentate gyrus (DG), nucleus accumbens (NAc)-core, NAc-shell, and striatum were captured using a 100× objective with a Keyence BZ-9000 GenerationⅡmicroscope (Osaka, Japan). Spine density in these regions was counted as previously reported (34, 36, 60). For spine density measurements, all clearly evaluable areas containing 50-100 µm of secondary dendrites from each imaged neuron were used. To determine relative spine density, spines on multiple dendritic branches from a single neuron were counted to obtain an average spine number per 10 µm. For spine number measurements, only spines that emerged perpendicular to the dendritic shaft were counted. Two to three neurons per section, three sections per animal were analyzed. The average value for each region, in each individual was obtained. These individual averages were then combined to yield a grand average for each region.

*Western blot analysis*

Western blot analysis was performed as reported previously (34, 36). Mice were killed by cervical dislocation and brains were rapidly removed from the skull. Approximately 1-mm-thick coronal sections were cut and bilateral tissue punches of PFC, NAc, CA1, CA3 and dentate gyrus (DG) of the hippocampus were dissected on ice using a SZ-LED Kenis light microscope (Osaka, Japan), and stored at -80°C. Basically, tissue samples were homogenized in Laemmli lysis buffer. Aliquots (10 μg) of protein were measured using the DC protein assay kit (Bio-Rad, Hercules, CA), and incubated for 5 min at 95 °C, with an equal volume of 125 mM Tris/HCl, pH 6.8, 20% glycerol, 0.1% bromophenol blue, 10% β-mercaptoethanol, 4% sodium dodecyl sulfate, and subjected to sodium dodecyl sulfate polyacrylamide gel electrophoresis, using 10% mini-gels (Mini-PROTEAN® TGX™ Precast Gel; Bio-Rad, CA, USA). Proteins were transferred onto polyvinylidenedifluoride (PVDF) membranes using a Trans Blot Mini Cell (Bio-Rad). For immunodetection, the blots were blocked with 2% BSA in TBST (TBS + 0.1% Tween-20) for 1 h at room temperature (RT), and kept with primary antibodies overnight at 4°C. The following primary antibodies were used: BDNF (1: 200, Santa Cruz Biotechnology, Inc., CA, USA), phosphor-TrkB (Tyr 706) (1:200, Santa Cruz Biotechnology, Inc., CA), TrkB (80E3) (1:1000, Cell Signaling Technology, MA), and AMPA glutamate receptor 1 (GluA1) (1 µg/ml, Abcam, Cambridge, MA). The next day, blots were washed three times in TBST and incubated with horseradish peroxidase conjugated anti-rabbit antibody (1:5000) 1 hour, at RT. After final three washes with TBST, bands were detected using enhanced chemiluminescence (ECL) plus the Western Blotting Detection system (GE Healthcare Bioscience). The blots then were washed three times in TBST and incubated with the primary antibody directed against β-actin (1:10000, Sigma-Aldrich Co, Ltd, St Louis, MO). Images were captured with a Fuji LAS3000-mini imaging system (Fujifilm, Tokyo, Japan), and immunoreactive bands were quantified.

*Parvalbumin (PV)-immunohistochemistry*

Saline (10 ml/kg), *R*-ketamine (10 mg/kg), or *S*-ketamine (10 mg/kg) was administered i.p. into mice. Thirty min after injection, mice were anesthetized with sodium pentobarbital (50 mg/kg) and perfused transcardially with 10 mL of isotonic saline, followed by 40 mL of ice-cold, 4% paraformaldehyde in 0.1%M phosphate buffer (pH 7.4). Brains were removed from the skulls and postfixed overnight at 4℃ in the same fixative. PV-immunohistochemistry was performed as previously reported (60). For the PV-immunohistochemistry, 50 μm-thick serial, coronal sections of brain tissue were cut in ice-cold, 0.001M phosphate buffered saline (pH 7.5) using a vibrating blade microtome (VT1000s, Leica Microsystems AG, Wetzlar, Germany). Free-floating sections were treated with 0.3% H_2_O_2_ in 50 mM Tris-HCL saline (TBS) for 30 min and were blocked in TBS containing 0.2% Triton X-100 (TBST) and 1.5% normal serum for 1 h at room temperature. The samples ware then incubated for 24 h at 4℃ with rabbit polyclonal anti-parvalbumin (PV) antibody (1:2,500, Swant, Bellinzona, Switzerland). The sections were washed three times in TBS and then processed using the avidin-biotin-peroxidase method (Vectastain Elite ABC, Vector Laboratories, Inc., Burlingame, CA, USA). Sections ware incubated for 3 min in a solution of 0.25 mg/mL DAB containing 0.01% H_2_O_2_. Then, sections were mounted on gelatinized slides, dehydrated, cleared, and cover slipped under Permount^Ⓡ^(Fisher Scientific, Fair Lawn, NJ, USA). The sections were imaged, and the staining intensity of PV immunoreactivity in the mPFC, hippocampus (CA1, CA3, DG) was analyzed using a light micro-scope equipped with a CCD camera (Olymups IX70) and the SCION IMAGE software package. Images of sections within mPFC and hippocampal CA1, CA3, DG regions were captured using a 100× objective with a Keyence BZ-9000 GenerationⅡmicroscope (Osaka, Japan).
